# Transient Receptor Potential Cation Channel Subfamily Vanilloid 4 and 3 in the Inner Ear Protect Hearing in Mice

**DOI:** 10.3389/fnmol.2019.00296

**Published:** 2019-12-04

**Authors:** Shengnan Wang, Qiaowei Geng, Lifang Huo, Yirui Ma, Yiting Gao, Wei Zhang, Hailin Zhang, Ping Lv, Zhanfeng Jia

**Affiliations:** ^1^Department of Pharmacology, Hebei Medical University, Shijiazhuang, China; ^2^Center for Innovative Drug Research and Evaluation, Institute of Medical Science and Health, Hebei Medical University, Shijiazhuang, China; ^3^The Key Laboratory of Neural and Vascular Biology, Ministry of Education, Shijiazhuang, China; ^4^The Key Laboratory of New Drug Pharmacology and Toxicology, Shijiazhuang, China; ^5^Department of Pharmacology, Institute of Chinese Integrative Medicine, Hebei Medical University, Shijiazhuang, China

**Keywords:** cochlear hair cell, TRPV, TRPV3, knockout, hearing

## Abstract

The transient receptor potential cation channel, vanilloid type (TRPV) 3, is a member of the TRPV subfamily that is expressed predominantly in the skin, hair follicles, and gastrointestinal tract. It is also distributed in the organ of Corti of the inner ear and colocalizes with TRPV1 or TRPV4, but its role in auditory function is unknown. In the present study, we demonstrate that TRPV3 is expressed in inner hair cells (HCs) but mainly in cochlear outer HCs in mice, with expression limited to the cytoplasm and not detected in stereocilia. We compared the number of HCs as well as distortion product otoacoustic emissions (DPOAE) and auditory brainstem response (ABR) thresholds between TRPV3 knockout (V3KO) and wild-type (V3WT) mice and found that although most mutants (72.3%) had normal hearing, a significant proportion (27.7%) showed impaired hearing associated with loss of cochlear HCs. Compensatory upregulation of TRPV4 in HCs prevented HC damage and kanamycin-induced hearing loss and preserved normal auditory function in most of these mice. Thus, TRPV4 and TRPV3 in cochlear HCs protect hearing in mice; moreover, the results suggest some functional redundancy in the functions of TRPV family members. Our findings provide novel insight into the molecular basis of auditory function in mammals that can be applied to the development of strategies to mitigate hearing loss.

## Introduction

Transient receptor potential (TRP) channels constitute a superfamily of non-selective cation channels that are involved in a variety of physiological processes (Clapham, 2003; Ramsey et al., 2006). TRP channels are divided into six subfamilies based on sequence homology–i.e., TRPC (classical), TRPV (vanilloid), TRPM (melastatin), TRPA (ankyrin), TRPP (polycystin), and TRPML (mucolipin). The TRPV subfamily comprises six members, TRPV1–TRPV6 (Clapham, 2003; Nilius and Szallasi, 2014). Four members of TRPV subfamily (TRPV1–TRPV4) are responded to temperature and referred to as thermosensitive TRPV (thermoTRPV) channels (Baez et al., 2014). These thermo TRPV channels can coassemble heteromeric channels with distinct conductance and gating properties from the homotetramer (Smith et al., 2002; Cheng et al., 2007).

TRPV3 differs in several aspects from other members of the TRPV subfamily (Nilius et al., 2014). For instance, TRPV3 is activated at an innocuous temperature (>33°C) unlike TRPV1 and TRPV2, which are activated by noxious heat (>43°C and >52°C, respectively; Peier et al., 2002; Smith et al., 2002; Xu et al., 2002; Nilius and Szallasi, 2014; Wang and Wang, 2017). *In vivo* studies have revealed deficits in response to innocuous and noxious heat in *Trpv3* knockout mice, whereas other sensory modalities were unaffected (Moqrich et al., 2005). TRPV3 is activated by several natural compounds such as carvacrol, eugenol, camphor, and thymol, as well as by the small synthetic compound 2-aminoethoxydiphenyl borate (Nilius and Szallasi, 2014; Wang and Wang, 2017). Unlike other thermos-TRPV channels, TRPV3 becomes sensitized rather than desensitized upon repeated stimulation with heat or agonists (Xu et al., 2002; Chung et al., 2004; Liu et al., 2011).

TRPV3 is most abundantly expressed in skin keratinocytes and in cells surrounding hair follicles, where it plays an essential role in cutaneous sensation including thermal sensation, nociception, and itch, in addition to maintenance of the skin barrier, wound healing, and hair growth (Peier et al., 2002; Imura et al., 2007; Cheng et al., 2010; Aijima et al., 2015). Gain-of-function mutations in human TRPV3 are associated with Olmsted syndrome, which is characterized by severe itch and palmoplantar and periorificial keratoderma (Lai-Cheong et al., 2012; Lin et al., 2012). In rodents, gain-of-function mutations of TRPV3 are associated with skin inflammation and pruritus (Asakawa et al., 2006; Yoshioka et al., 2009). On the other hand, itching behavior is suppressed in TRPV3 knockout mice (Yamamoto-Kasai et al., 2012). Besides the skin, TRPV3 is expressed in various neuronal and non-neuronal tissues, suggesting that it has important roles in cellular and physiological functions (Luo and Hu, 2014; Nilius and Szallasi, 2014).

TRPV channels are expressed in inner ear tissues in vertebrates, and some are presumed to be involved in hearing (Zanini and Göpfert, 2014). For example, TRPV4 is present in hair cells (HCs) and adjacent supporting cells of the organ of Corti, marginal cells of the stria vascularis, and ganglion neurons (Ishibashi et al., 2008). The *Trpv4* gene is associated with *Deafness, autosomal dominant, 25*, a locus that is linked to dominant nonsyndromic hereditary hearing impairment (Greene et al., 2001). TRPV4 mutation in humans results in mild to moderate progressive hearing loss (Oonk et al., 2014), while TRPV4 knockout mice show delayed-onset hearing loss (Tabuchi et al., 2005). TRPV1 is expressed in HCs and supporting cells of the organ of Corti (Zheng et al., 2003; Ishibashi et al., 2008) and facilitates cochlear uptake of aminoglycosides; bacteriogenic stimulation up-regulates TRPV1 expression to exacerbate cochleotoxicity (Jiang et al., 2019). Additionally, loss-of-function polymorphisms in *Trpv1* protect against immunogenic exacerbation of kanamycin-induced HC and hearing loss (Jiang et al., 2019). TRPV3 is expressed in the organ of Corti and often colocalizes with TRPV1 or TRPV4 (Ishibashi et al., 2008). However, its function in the inner ear is unknown.

In the present study, we examined TRPV3 expression in the HCs of mice and investigated the effect of TRPV3 loss on auditory thresholds using TRPV3 knockout (V3KO) mice. We found that a significant fraction (30%) of these mice showed impaired hearing, which was accompanied by a reduction in HC number, while 70% of V3KO mice had normal hearing. Moreover, we observed a compensatory upregulation of TRPV4 in HCs in response to TRPV3 deficiency to maintain their normal hearing and protect against kanamycin-induced ototoxicity.

## Materials and Methods

### Animals

V3KO mice were provided by Professor Kewei Wang at the College of Pharmacy, Qingdao University. The mice were produced and maintained on a C57BL/6 wild-type (WT) background and were genotyped by PCR using the following primers: TRPV3 (standard forward primer), GGCCCTCAGAGGAGCC; V3WT-R, CAGGTACTGTGTCGCCCC (WT-specific reverse primer); and V3KO-R, TCTATGGCTTCTGAGGCGG (mutant-specific reverse primer). Genomic DNA was isolated from mouse tails, and PCR amplification was performed as previously reported (Jörs et al., 2010; Zhang et al., 2018).

Male and female V3KO mice aged 2–3 months with bodyweight between 17 and 25 g were used for experiments. Sex- and age-matched WT TRPV3 (V3WT) mice served as controls. The mice were housed at room temperature (22°–24°C) with free access to food/water on a 12:12-h light/dark cycle. Experimental procedures were approved by the Animal Care and Use Committee of Hebei Medical University.

### Kanamycin Administration

Kanamycin was purchased from Beijing Brinway Technology Co. (Beijing, China). V3WT and V3KO mice (*n* = 10 each) were subcutaneously injected with kanamycin sulfate at 1,000 mg/kg twice daily for 2 weeks (Jansen et al., 2013). Another group of V3WT mice (*n* = 10) was injected with an equal volume of saline. Auditory brainstem response (ABR) thresholds in response to clicks and 3-ms pure tones were measured before and 2 weeks after kanamycin administration.

### Measurement of Auditory Brainstem Response (ABR)

ABR threshold was measured as previously described (Shen et al., 2018) using a System III workstation (Tucker Davis Technologies, Alachua, FL, USA) in an IAC BioSigRP Soundbooth (GM Instruments, Irvine, UK). Briefly, mice were anesthetized by intraperitoneal injection of ketamine (100 mg/kg) and xylazine (10 mg/kg). Subcutaneous electrodes were placed at the vertex (reference) and behind the right ear (active), and a ground electrode was placed in the back. ABRs were measured in response to tone pips of 4, 8, 12, 16, 20, 24, 28, and 32 kHz, and the auditory threshold was determined by decreasing the sound intensity from 90 to 20 dB until the lowest sound intensity at which reproducible waves could be recognized was reached.

### Measurement of Distortion Product Otoacoustic Emission (DPOAE)

Mice were anesthetized as described above, and the distortion product otoacoustic emission (DPOAE) threshold was measured as previously described (Bautista et al., 2006). Briefly, f1 and f2 primary tones generated by a two-channel frequency synthesizer were presented over two Realistic tweeters (Optimus, Vancouver, BC, Canada) and delivered through a soft rubber probe. Ear canal sound pressure was measured with a commercial 10B+ acoustic probe (Etymotic Research, Elk Grove Village, IL, USA). To measure DPOAE, the generator was directly inserted into the ear canal, and closed-field detection was performed at frequencies of 4, 8, 16, 28, and 32 kHz on a System III workstation in an IAC BioSigRP Soundbooth.

### Immunofluorescence Analysis

Mice were transcardially perfused with 4% paraformaldehyde (PFA) under terminal anesthesia (sodium pentobarbital, 80 mg/kg), and the cochlea was dissected from the temporal bone and fixed overnight at 4°C in 4% PFA. The samples were sequentially processed with 10% EDTA, followed by 10% and 30% sucrose overnight at 4°C, then embedded in Optimal Cutting Temperature compound for cryosectioning.

Cochlear sections were cut at a thickness of 10 μm, washed once with PBS, permeabilized for 30 min with 0.3% Triton X-100 in PBS, and blocked for 60 min with 3% bovine serum albumin (BSA) at 37°C. The sections were incubated overnight at 4°C with primary antibody diluted in 0.1% Triton X-100 solution containing 1% BSA. The following primary antibodies were used: rabbit myosin-VIIa (Bioscience, Allentown, PA, USA), mouse myosin-VIIa (Santa Cruz Biotechnology, Santa Cruz, CA, USA), rat phalloidin-iFluor 555 (Abcam, Cambridge, MA, USA), mouse or rabbit anti-TRPV3 antibody (Genentech, San Francisco, CA, USA), mouse anti-TRPV1 antibody and rabbit anti-TRPV4 antibody (Alomone Labs, Jerusalem, Israel). Sections were rinsed with PBS, then incubated for affinity-purified h at room temperature with fluorescein isothiocyanate-conjugated affinity-purified goat anti-rabbit IgG and Cy3-conjugated affinity-purified goat anti-mouse IgG as secondary antibodies (Jackson Laboratories, Bar Harbor, ME, USA). Nuclei were labeled with 4′,6-diamidino-2-phenylindole (DAPI). Images anti-rabbit with a TCS SP5 confocal microscope (Leica, Wetzlar, Germany).

For the preparation of cochlear HC tissue, the spiral ligament/lateral wall of cochlear samples was removed after decalcification, and a portion of cochlear HCs was retained. IF labeling and visualization of HC tissues were performed as described above.

### Statistical Analysis

Data are presented as mean ± SEM and were analyzed with SPSS software (SPSS Inc., Chicago, IL, USA). The significance of differences between groups was evaluated with the Student’s *t*-test or by one-way analysis of variance followed by Dunnett’s *post hoc* test for multiple groups. The *χ*^2^ test was used to assess differences in hearing impairment between V3WT and V3KO mice. *P* < 0.05 was considered statistically significant.

## Results

### TRPV3 Expression in the Inner Ear

**Figure 1:**
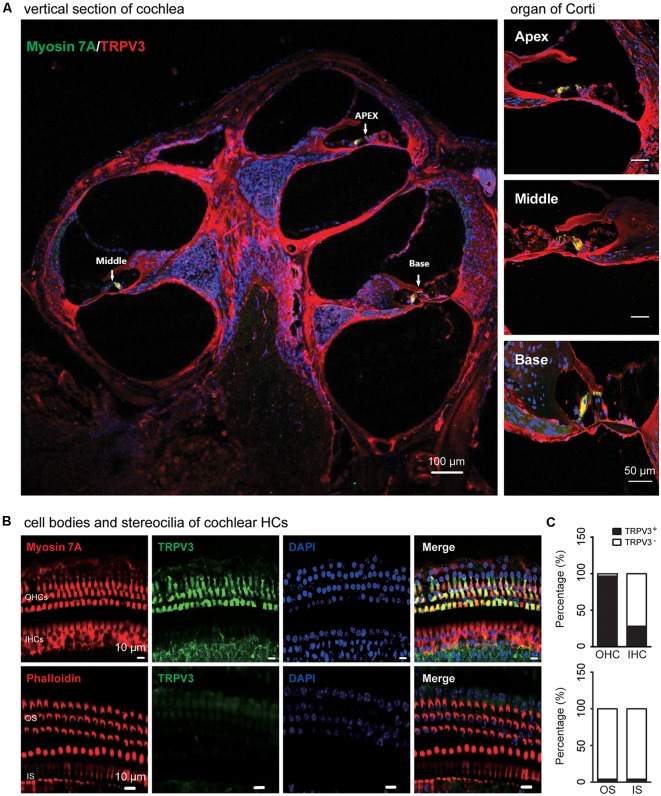
Immunolabeling of transient receptor potential cation channel, vanilloid type (TRPV) 3 in the cochlea of mice. **(A)** Representative images of TRPV3 expression (red) in cochlear sections are shown (left panel). The apex, middle, and base of the cochlea (arrow) are expanded from the left panel and presented in the upper, middle, and bottom-right panels, respectively. Cell bodies (myosin 7A, green) and cell nuclei 4′,6-diamidino-2-phenylindole (DAPI, blue) of hair cells (HCs) are labeled. Scale bars = 100 μm (left panels) and 50 μm (right panels). **(B)** Immunolabeling of TRPV3 (green) in cell bodies (myosin 7A, red) and stereocilia (phalloidin, red) of HCs in cochlear preparations; cell nuclei are stained with DAPI (blue). Scale bars = 10 μm. **(C)** Percentage of TRPV3-positive (TRPV3^+^, solid bar) and -negative (TRPV3^−^, open bar) HCs (upper panels), and percentage of TRPV3-positive and -negative stereocilia (bottom panels) in cochlear preparations.

**Figure 2:**
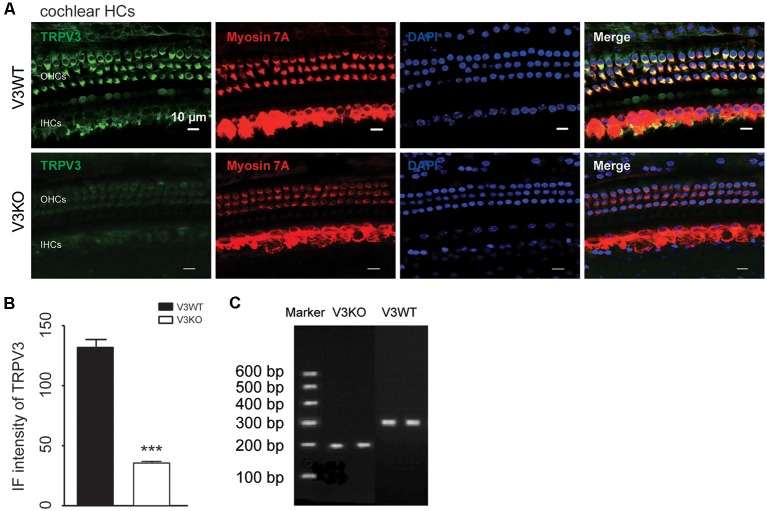
Immunolabeling of TRPV3 in cochlear HCs in V3WT and V3KO mice. **(A)** Representative images of TRPV3 (green) expression in outer HCs (OHCs) and inner HCs (IHCs) in cochlear preparations from V3WT and V3KO mice, respectively. Cell bodies (myosin 7A) and cell nuclei (DAPI, blue) of cochlear HCs in V3WT and V3KO mice are shown. Scale bars = 10 μm. **(B)** Immunofluorescence (IF) intensity of TRPV3 in cochlear OHCs of V3WT (solid bar) and V3KO (open bar) mice. **(C)** Genotyping results showing *Trpv3* gene knockout in mice. Data are shown as mean ± SEM. ***P < 0.001.

TRPV3 expression in the inner ear was examined by IF labeling. Myosin-VIIa (7A), which is primarily expressed in the inner ear (Walters et al., 2017), has been used to selectively label HCs of the organ of Corti (Figures 1A,B). Stereocilia were labeled with phalloidin (Chanda et al., 2018), a fluorescent probe targeting actin filaments in HC cilia (Figure 1B). HC preparations exhibited a regular pattern of three outer HC (OHC) rows and a single row of inner HCs (IHCs; Figure 1B). All OHCs had normal nuclei and stereocilia bundles (Figure 1B). TRPV3 was predominantly expressed in HCs of the organ of Corti in the apical, middle, and basal turns of the cochlea (Figure 1A), whereas no expression was detected in the stria vascularis or spiral ganglion (SG) neurons (Figure 1A). TRPV3 showed distinct expression patterns in OHCs and IHCs: the protein was detected in 96.8% of OHCs (*n* = 500) and 28% of IHCs (*n* = 200; Figures 1B,C). This indicates that TRPV3 is mainly present in OHCs of the organ of Corti in mice. Cell bodies of HCs showed strong TRPV3 IF, but there was almost no signal in inner or outer stereocilia (IS and OS, respectively; OS, *n* = 500; IS, *n* = 150; Figures 1B,C). Thus, TRPV3 localizes to the cell body and not the stereocilia of HCs. *Trpv3* knockout (Figure 2C) reduced TRPV3 expression in HCs (Figures 2A,B), as evidenced by the signal intensity of 35 arbitrary units (A.U.; *n* = 500) compared to 130 A.U. in V3WT mice (*n* = 500; *P* < 0.001).

### Function of TRPV3 in Hearing

**Figure 3:**
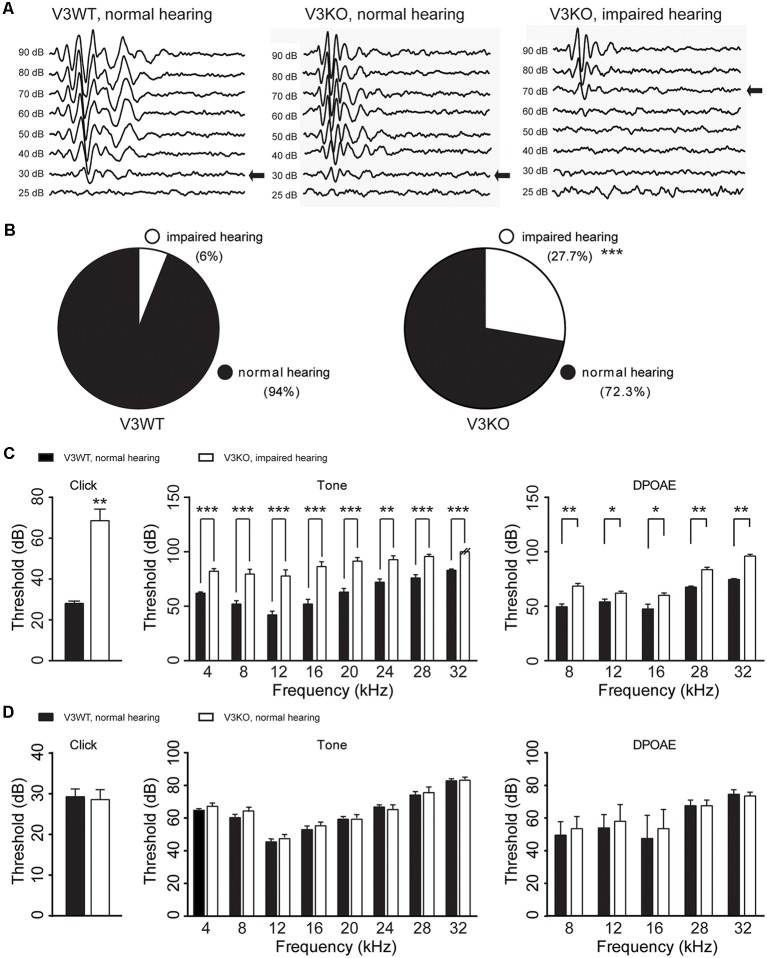
Hearing in V3WT and V3KO mice. **(A)** Representative auditory thresholds (bold arrow) were measured in V3WT (left) and V3KO mice with normal hearing (middle) and V3KO mice with impaired hearing (right). **(B)** The proportion of mice with impaired (open circle) and normal (solid circle) hearing in the V3WT (left) and V3KO (right) groups. **(C)** Auditory brainstem response (ABR) thresholds in response to broadband clicks (left) and 3-ms pure tones of 4, 8, 12, 16, 20, 24, 28, and 32 kHz (middle) and DPOAE thresholds (right) measured in V3WT mice with normal hearing (solid bar) and V3KO mice with impaired hearing (open bar). **(D)** Click (left), tone (middle), and DPOAE (right) thresholds measured in V3WT (solid bar) and V3KO mice both with normal hearing (open bar). Data are shown as mean ± SEM (*n* = 10 per group). *P < 0.05, **P < 0.01, ***P < 0.001.

To investigate the role of TRPV3 in hearing, ABR was measured and compared in V3KO and V3WT mice. We found that 94% (47/50) V3WT mice had normal hearing, and only 6% (3/50) showed spontaneous, nonspecific hearing loss (Figures 3A,B). In contrast, 27.7% (15/54) of V3KO mice had hearing impairment (*P* < 0.001), although in 72.3% (39/54), hearing was normal (Figures 3A,B). In hearing-impaired V3KO mice, the threshold in the click test was 70 dB compared to 30 dB in V3WT mice (Figure 3C). The threshold in the pure tone test was also higher in hearing-impaired V3KO mice than in V3WT mice at all tested frequencies—i.e., 80 vs. 65 dB at 4 kHz (*P* < 0.001), 90 vs. 70 dB at 20 kHz (*P* < 0.001), and >90 (testing limit) vs. 80 dB at 32 kHz (*P* < 0.001; Figure 3C).

To examine OHC function *in vivo*, we compared DPOAE in V3WT and V3KO mice and found that the threshold was increased in hearing-impaired V3KO mice at the tested frequencies (Figure 3C). However, there were no significant differences in thresholds in the ABR and DPOAE tests between V3WT mice and V3KO mice with normal hearing (Figure 3D). These results indicate that auditory function varies in the absence of TRPV3 from loss of hearing to normal hearing.

### Compensatory Upregulation of TRPV4 Protects Against HC Impairment and Hearing Loss in V3KO Mice

**Figure 4:**
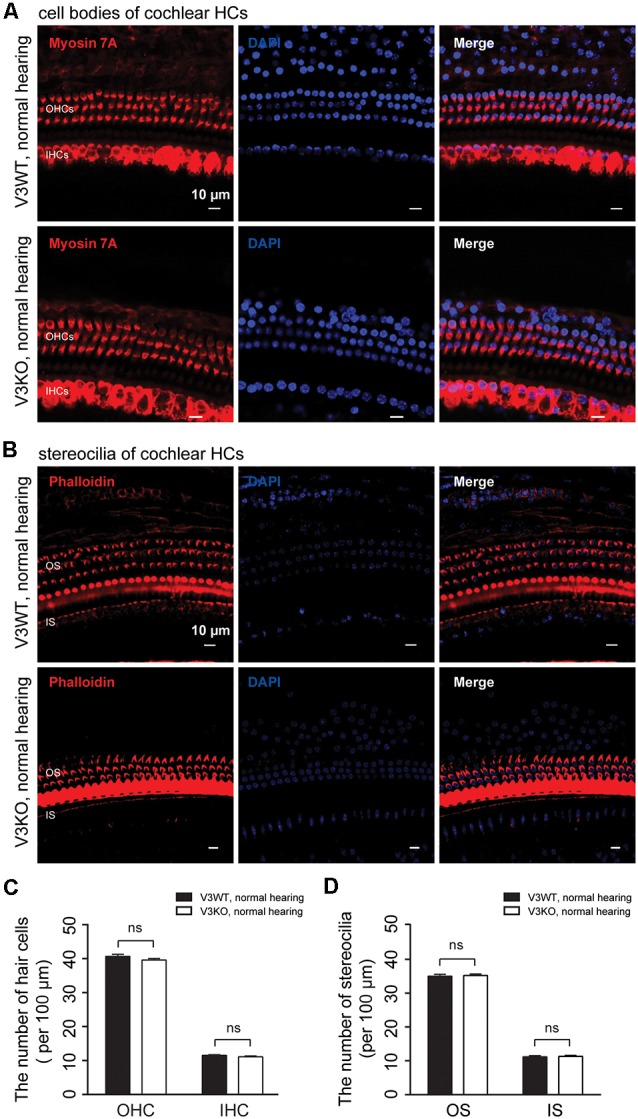
HC patterns in cochlear preparations from V3WT and V3KO mice with normal hearing. **(A)** Cell bodies (myosin 7A, red) and nuclei (DAPI, blue) of OHCs in a three-row pattern and IHCs in a single-row pattern in cochlear preparations from V3WT (upper panels) and V3KO (bottom panels) mice with normal hearing are shown. Scale bars = 10 μm. **(B)** Outer stereocilia (OS) stained with phalloidin (red) and cell nuclei stained with DAPI (blue) in OHCs in a three-row pattern and inner stereocilia (IS) in a single-row pattern in cochlear HCs from V3WT (upper) and V3KO (bottom) mice with normal hearing. Scale bars = 10 μm. Quantitative analysis of cochlear HCs **(C)** and stereocilia **(D)** per 100 μm in cochlear preparations of V3WT (solid bar) and V3KO (open bar) mice with normal hearing. Data are shown as mean ± SEM. ns, non-significant.

To investigate the mechanistic basis for the observed differences in hearing function among V3KO mice, we first examined the morphology of HCs and their stereocilia by IF labeling in V3WT mice and V3KO mice with normal hearing (Figure 4). The three rows of OHCs and their stereocilia were observed in all of the mice (Figures 4A–D), and a quantitative analysis revealed that there was no loss of HCs in V3KO mice [OHCs: 40 ± 3 per 100 μm (*P* > 0.05) and IHCs: 12 ± 1 per 100 μm (*P* > 0.05)] relative to V3WT mice (OHCs: 40 ± 2 per 100 μm and IHCs: 11 ± 1 per 100 μm; Figures 4A,C). The number of stereocilia was also comparable between V3WT mice and V3KO mice with normal hearing (Figures 4B,D). These results indicate that the loss of TRPV3 does not impair cochlear HCs.

**Figure 5:**
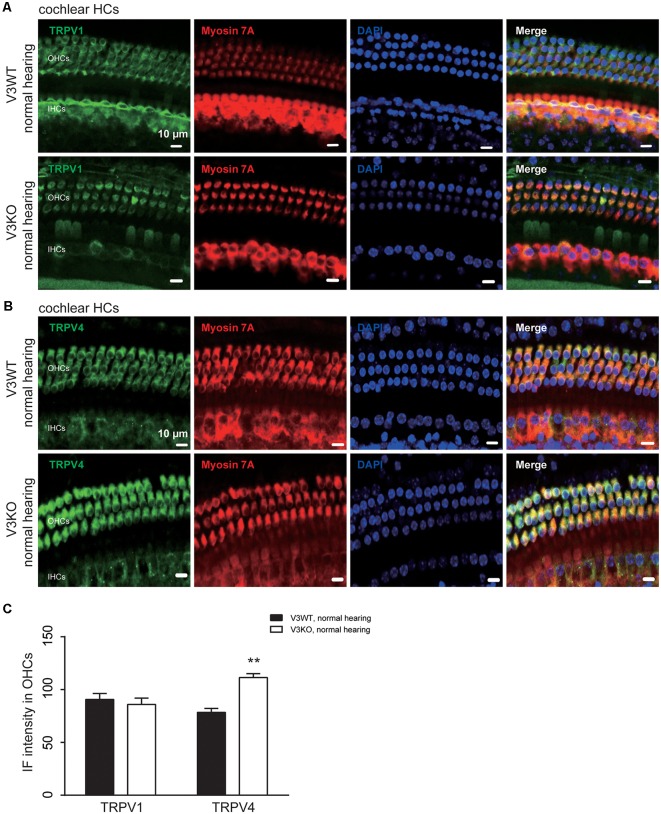
Immunolabeling of TRPV1 and TRPV4 in cochlear preparations of V3KO mice with normal hearing. **(A,B)** Representative images of TRPV1 (green; **A**) and TRPV4 (green; **B**) in HCs from the base turn of cochlear preparations from V3WT and V3KO mice with normal hearing. Cell bodies (myosin 7A, red) and cell nuclei (DAPI, blue) of OHCs in a three-row pattern and IHCs in a single-row pattern are shown. Scale bars = 10 μm. **(C)** IF intensity of TRPV1 and TRPV4 in cochlear OHCs of V3WT (solid bar) and V3KO (open bar) mice with normal hearing. Data are shown as mean ± SEM (*n* = 5 per group). **P < 0.01.

Previous studies have shown that TRPV1 and TRPV4 in HCs of the organ of Corti play an important role in the maintenance of normal hearing (Zheng et al., 2003; Tabuchi et al., 2005; Żak et al., 2016). We investigated whether TRPV1 or TRPV4 expression is altered in V3KO mice with normal hearing. Similar to TRPV3, TRPV1 and TRPV4 were mainly detected in OHCs, with no apparent differences in TRPV1 expression between V3WT mice and V3KO mice with normal hearing (Figures 5A,C). On the other hand, TRPV4 was upregulated in HCs in the latter compared to the former group (Figures 5B,C): the IF intensity value of TRPV4 in HCs of V3KO mice (*n* = 50) with normal hearing was 117 A.U. as compared to 78 A.U. in V3WT mice (*n* = 50; Figure 5C). Thus, loss of TRPV3 leads to a compensatory upregulation of TRPV4 but not TRPV1 in HCs, thereby protecting against HC and hearing loss.

**Figure 6:**
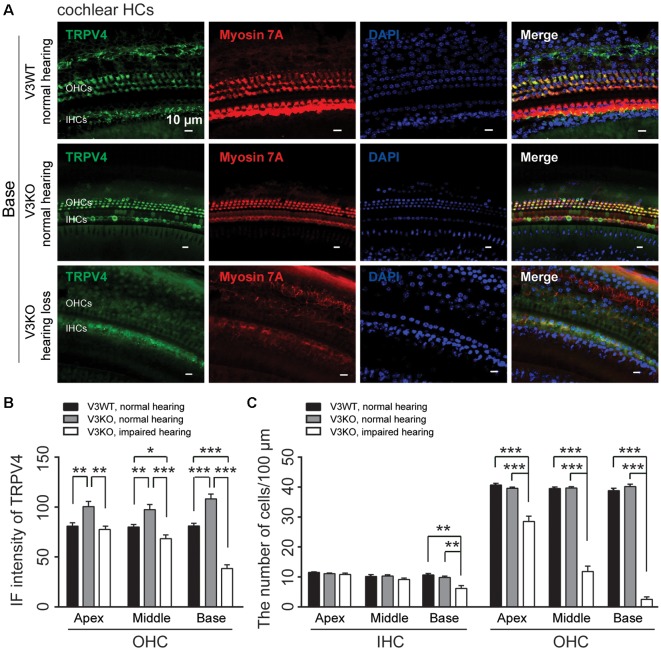
Immunolabeling of TRPV4 in HCs in cochlear preparations of V3KO mice with impaired hearing. **(A)** Representative images of TRPV4 (green) in HCs from the base turn of cochlear preparations in V3WT (top) and V3KO (middle) mice with normal hearing and V3KO mice with impaired hearing (bottom). Cell bodies (myosin 7A, red) and cell nuclei (DAPI, blue) are shown. Scale bars = 10 μm. **(B)** IF intensity of TRPV4 in the apex, middle, and base turn of cochlear OHCs from V3WT (solid bar) and V3KO (gray bar) mice with normal hearing and V3KO mice with impaired hearing (open bar). **(C)** Quantitative analysis of the number of cochlear HCs per 100 μm in the apex, middle, and base of cochlea samples from the above three groups. Data are shown as mean ± SEM (*n* = 5 per group). *P < 0.05, **P < 0.01, ***P < 0.001.

To clarify the mechanistic basis for the above observation, we examined TRPV4 expression in HCs of V3KO mice with impaired hearing by immunolabeling. TRPV4 expression in OHCs of the middle and basal cochlea is significantly down-regulated in hearing-impaired V3KO mice (Figures 6A,B): the IF intensity was 66 A.U. in OHCs of the middle region of the cochlea in hearing-impaired V3KO mice, which was significantly lower than the values of 80 A.U. in V3WT mice (*P* < 0.05) and 98 A.U. in V3KO mice with normal hearing (*P* < 0.001; Figure 6B). In OHCs of the basal cochlea, the IF intensity of TRPV4 was 38 A.U. in hearing-impaired V3KO mice as compared to 81 A.U. in V3WT mice (*P* < 0.001) and 108 A.U. in V3KO mice with normal hearing (*P* < 0.001; Figures 6A,B). These results indicate that TRPV4 expression is decreased to a greater extent in OHCs of the basal as compared to the middle cochlea in hearing-impaired V3KO mice. We also quantified HCs in hearing-impaired V3KO mice and found that the number of IHCs in the basal region of the cochlea was lower (7 per 100 μm) than in V3WT mice (10.7 per 100 μm; *P* < 0.01) and V3KO mice with normal hearing (9.8 per 100 μm; *P* < 0.01; Figure 6C). Thus, the loss of OHCs was more significant in hearing-impaired V3KO mice. OHC numbers were decreased in all three parts of the cochlea—the apex, middle, and base (Figure 6C). For example, the number of OHCs in the basal region was 3 per 100 μm in hearing-impaired V3KO mice, as compared to 39 per 100 μm in V3WT mice (*P* < 0.001) and 40 per 100 μm in V3KO mice with normal hearing (*P* < 0.001; Figure 6C). Thus, *Trpv3* deficiency and downregulation of TRPV4 results in a significant loss of HCs, which impairs hearing.

### Compensatory Upregulation of TRPV4 Expression in the Absence of TRPV3 Protects Against Kanamycin-Induced Ototoxicity

**Figure 7:**
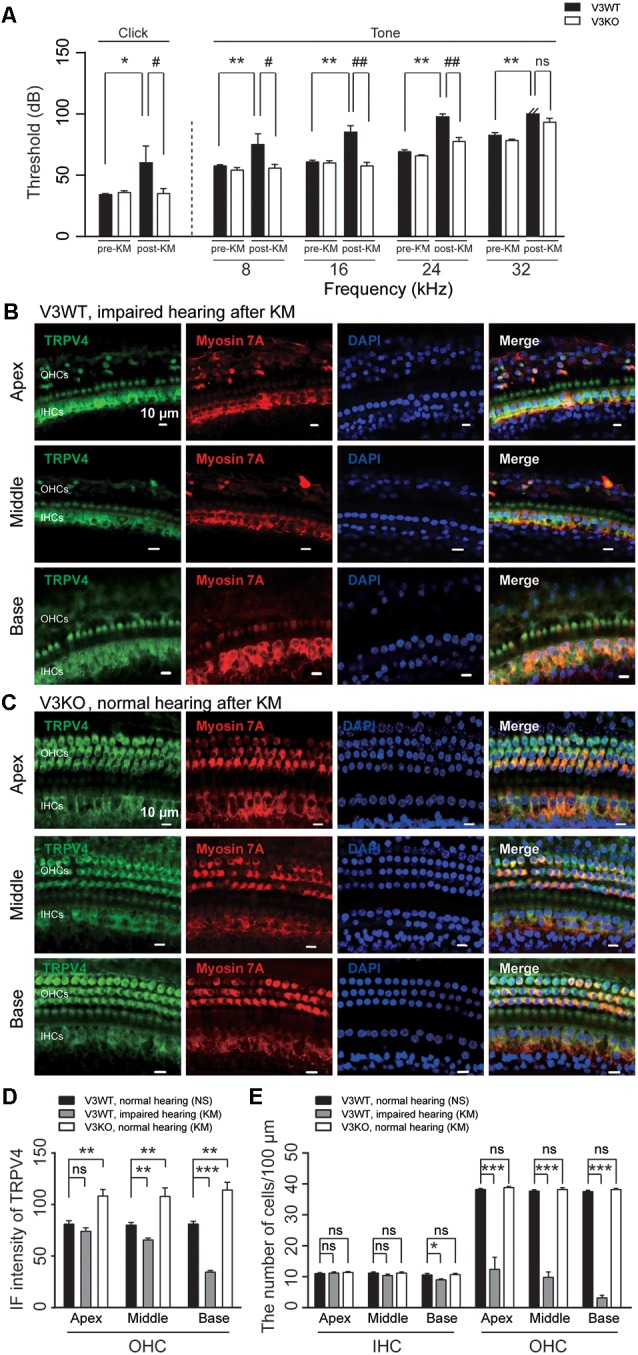
The upregulation of TRPV4 protects V3KO mice against kanamycin challenge. **(A)** ABR thresholds in response to click and 3-ms pure tones of 8, 16, 24, and 32 kHz were measured in V3WT (solid bar) and V3KO (open bar) mice before (pre-KM) and after (post-KM) kanamycin administration (*n* = 10 per group). **(B,C)** Immunolabeling of TRPV4 (green) in the apex, middle, and base of the cochlea from V3WT mice with impaired hearing and V3KO mice with normal hearing after kanamycin treatment. Cell bodies (myosin 7A, red) and nuclei (DAPI, blue) of cochlear HCs are shown. Scale bars = 10 μm. **(D)** IF intensity of TRPV4 in cochlear OHCs from V3WT mice with normal hearing after saline treatment (NS, solid bar), V3WT mice with impaired hearing after kanamycin (KM) treatment (gray bar), and V3KO mice with normal hearing after kanamycin treatment (open bar). **(E)** Quantitative analysis of the number of IHCs and OHCs per 100 μm in apex, middle, and base of cochlea from the above three groups. Data are shown as mean ± SEM (*n* = 10 per group). *^#^*P* < 0.05, **^##^*P* < 0.01, ***P < 0.001; ns, non-significant.

TRPV4 plays a vital role in auditory function (Tabuchi et al., 2005). Aminoglycoside inhibits TRPV4 expression in HCs (Ishibashi et al., 2009). In this study, loss of TRPV3 resulted in compensatory upregulation of TRPV4 expression in cochlear HCs, which preserved normal hearing in most V3KO mice. To clarify the significance of this observation to auditory function, ABR was measured, and TRPV4 expression was evaluated in V3WT mice and V3KO mice with normal hearing before and after kanamycin treatment. There was no difference in auditory threshold in the click and tone tests between groups before kanamycin treatment. However, after kanamycin challenge, the auditory threshold in the click test was increased from 30 to 70 dB in V3WT but not V3KO mice (*P* < 0.05; Figure 7A). Surprisingly, in the absence of TRPV3 the auditory threshold in the click test remained at 35 dB even after kanamycin treatment and differed from the value measured in V3WT mice (*P* < 0.05; Figure 7A). A similar phenomenon was observed in the tone test, with kanamycin treatment increasing the auditory threshold at all tested frequencies (8, 16, 24, and 32 kHz) in V3WT but not in V3KO mice (Figure 7A). For example, at 8 kHz, V3WT mice treated with kanamycin showed an increased threshold (from 55 to 75 dB; *P* < 0.01), whereas that in V3KO mice was unchanged (*P* > 0.05; Figure 7A). At 24 kHz, kanamycin caused the auditory threshold to exceed 90 dB in V3WT mice (*P* < 0.01), while that in V3KO mice remained at 75 dB (*P* < 0.01; Figure 7A). Immunolabeling experiments showed that TRPV4 expression in OHCs decreased gradually along the apical, middle, and basal cochlea in kanamycin-treated hearing-impaired V3WT mice (Figures 7B,D). Furthermore, the spatial patterning of OHCs but not IHCs in the organ of Corti was severely disrupted in V3WT mice following kanamycin treatment (Figures 7B,E). Interestingly, TRPV4 expression in OHCs of the organ of Corti increased rather than decreased in V3KO mice with normal hearing upon kanamycin treatment compared to control V3WT mice (Figures 7C,D) and the number and patterning of both OHCs and IHCs were preserved (Figures 7C,E). These results indicate that compensatory upregulation of TRPV4 in the absence of TRPV3 protects against kanamycin-induced ototoxicity.

## Discussion

**Figure 8:**
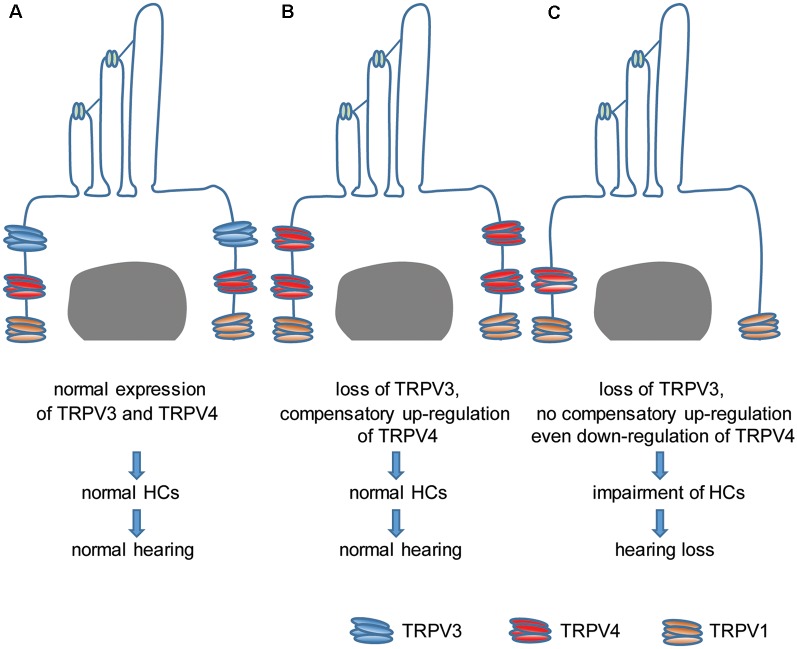
Model of the role of TRPV3 and TRPV4 in cochlear HCs in hearing. **(A)** Normal expression of TRPV3 and TRPV4 in cochlear HCs is critical for maintaining a normal number of HCs and normal hearing. **(B)** The loss of TRPV3 leads to compensatory upregulation of TRPV4 in cochlear HCs, which preserves the HC population and normal hearing. **(C)** Failure of compensatory upregulation or downregulation of TRPV4 in cochlear HCs under TRPV3 deficiency leads to cochlear impairment and hearing loss.

This study investigated the relationship between TRPV3 and hearing. We found that TRPV3 is mainly expressed in OHCs of the organ of Corti in mice and that its deficiency results in HC damage and hearing loss in approximately 30% of mice, whereas the remaining 70% retain normal hearing. This was likely due to the compensatory upregulation of TRPV4, which even protected mice against aminoglycoside-induced ototoxicity. Our results indicate that normal expression of TRPV3 and TRPV4 in HCs is important for the maintenance of normal HC morphology and auditory function. Failure of compensatory upregulation of TRPV4 may result in HC impairment and hearing loss (Figure 8). This is the first description of a role for TRPV3 in auditory function and the underlying mechanism of action.

TRPV subfamily members are expressed in the inner ear of mammals and play an important role in hearing. TRPV1, TRPV2, TRPV3, and TRPV4 are co-expressed in HCs and supporting cells of the organ of Corti, SG neurons, vestibular sensory cells, and vestibular ganglion (VG) and trigeminal ganglion neurons (Kitahara et al., 2005; Ishibashi et al., 2008). TRPV4 is detected in the stria vascularis (Liedtke et al., 2000; Kitahara et al., 2005; Ishibashi et al., 2008, 2009). TRPV1 is highly expressed in SG and VG neurons, whereas TRPV4 is downregulated in these cells following the kanamycin challenge (Kitahara et al., 2005). Expression of TRPV1 and TRPV2 was increased whereas that of TRPV3 and TRPV4 was decreased in the inner ear after gentamicin treatment (Ishibashi et al., 2009). Based on these observations, it was suggested that TRPV3 and TRPV4 play an important role in neuroprotection while TRPV1 and TRPV2 are involved in the pathology of hearing loss (Ishibashi et al., 2009). Consistent with previous studies (Ishibashi et al., 2009), we detected TRPV3 expression in HCs along the apex, middle, and base of the cochlea but not in stria vascularis cells. Furthermore, TRPV3 was mainly expressed in OHCs but not IHCs of the organ of Corti. However, we did not observe TRPV3 in SG neurons of the cochlea. Our results suggest that TRPV3 is important for maintaining HCs and hearing under normal conditions in the mouse. TRPV4 may play a more significant role in this process than TRPV3, as implied by the observation that a loss-of-function mutation in human TRPV4 or TRPV4 knockout in mice resulted in progressive hearing loss (Tabuchi et al., 2005; Oonk et al., 2014).

TRPV3 and other TRP channels are unlikely to function as auditory transduction channels in HCs (Kazmierczak and Müller, 2012; Zanini and Göpfert, 2014), given the lack of evidence that they are mechanically gated (Delmas et al., 2011). Other proteins such as Tetraspan membrane protein of HC stereocilia (Xiong et al., 2012), Transmembrane channel-like protein (TMC) 1 and TMC2 (Kawashima et al., 2011; Pan et al., 2013), Transmembrane inner ear expressed gene (Zhao et al., 2014), and Piezo2 (Wu et al., 2017) have been proposed as essential components of mechanotransduction channels in cochlear HCs. As shown in our work, protecting HCs from pathological impairment may be the major function of TRPV3 and TRPV4 in hearing. The upregulation of TRPV4 may compensate for the deficiency of TRPV3 to keep the normal appearance of HCs and even resist kanamycin-induced HC impairment. However, the precise mechanism by which TRPV3 deficiency leads to the upregulation of TRPV4 in cochlear HCs remains to be determined. Our results also contradict previous findings: Tabuchi et al. (2005) have demonstrated that TRPV4 knockout mice do not exhibit any HC loss, although their hearing was impaired. It is not known how TRPV4 protects cochlear HCs. Evidence has been demonstrated that extracellular hypoosmotic stimulation-induced Ca^2+^ influx in OHCs (Harada et al., 1994). TRPV4, functions as an osmotic sensor (Liedtke et al., 2000), may be involved in this process. It is well known that Ca^2+^ homeostasis plays a crucial role in cell fate determination. Ca^2+^ influx may induce intracellular Ca^2+^ overload in OHCs and hence cause HC death (Fettiplace and Nam, 2019). Thus, it is unlikely that TRPV4 protects HCs by maintaining Ca^2+^ homeostasis since channel activation cannot prevent Ca^2+^ overload under physiological conditions.

Uptake of aminoglycoside antibiotics, including kanamycin, gentamicin, and neomycin, in the inner ear HCs leads to HC death and irreversible hearing loss (Forge and Schacht, 2000; Jiang et al., 2017). Aminoglycosides predominantly enter inner ear HCs by permeating the mechanoelectrical transduction channel, TMC1 and TMC2 (Marcotti et al., 2005; Kawashima et al., 2011; Pan et al., 2018), non-selective cation channels including TRPV channels (Karasawa et al., 2008; Stepanyan et al., 2011; Jiang et al., 2019), as well as *via* endocytosis (Hashino and Shero, 1995). As far as TRPV channels, TRPV1 has been well studied on its role in mediating aminoglycosides uptake in HCs (Jiang et al., 2017). TRPV1 is expressed in the stria vascularis and HCs (Zheng et al., 2003; Mukherjea et al., 2011), the critical locations in the trafficking of aminoglycosides from the bloodstream into sensory HCs (Li and Steyger, 2011). TRPV1 facilitates cochlear HCs uptake of aminoglycosides, and bacteriogenic stimulation upregulates its expression to exacerbate cochleotoxicity (Jiang et al., 2019). Similar to TRPV1, TRPV4 is also gentamicin permeant (Karasawa et al., 2008; Lee et al., 2013), but it is downregulated in inner ear ganglia following chronic kanamycin treatment (Kitahara et al., 2005). Combined with this study, compensatory upregulation TRPV4 prevents kanamycin-induced HCs damage and hearing loss in V3KO mice. Thus, we wonder whether TRPV4 has a significant difference in the uptake of gentamicin and kanamycin even TRPV4 prevents the absorption of kanamycin in HCs. Further investigation for the HCs uptake of kanamycin through TRPV4, as well as the mechanisms underlying protection of HCs by TRPV4 or TRPV3, is required.

Similar to V3KO mice, TRPA1 knockout mice have normal hearing (Bautista et al., 2006; Kwan et al., 2006). It has been suggested that functionally redundant proteins compensate for the loss of TRPA1 in the inner ear. Given that TRPA1 and TRPV1 reportedly interact and mutually regulate (Akopian et al., 2007; Fischer et al., 2014; Weng et al., 2015), upregulation of TRPV1 may offset TRPA1 deficiency, although direct supporting evidence for this is lacking (Asai et al., 2010). TRPV3 co-assembles into a heterotetramer with TRPV1, TRPV2, or TRPV4 (Cheng et al., 2007). However, TRPV1 does not appear to play a role in the effects of TRPV3 deficiency, given that its expression in cochlear HCs did not differ between V3WT and V3KO mice. This is consistent with the fact aforementioned that TRPV1 plays a pathological role in auditory function. In this context, the notion that the upregulation of TRPV1 compensates for TRPA1 deficiency to preserve normal hearing is unreasonable. Our findings suggest one possibility that increased expression of TRPV4 mitigates TRPA1 deficiency to promote the maintenance of normal hearing. However, additional studies are required to examine the interaction and regulation of TRPA1 and TRPV4 in greater detail.

In summary, the results of this study strongly demonstrate that members of the TRPV subfamily—especially TRPV4 and TRPV3—in cochlear HCs protect hearing in mice. Additionally, the up-regulation of TRPV4 expression may compensate for TRPV3 deficiency to maintain normal hearing and protect against kanamycin-induced ototoxicity. These findings provide novel insight into the molecular basis of auditory function in mammals that can be used to develop strategies to mitigate hearing loss in humans.

## Data Availability Statement

All datasets generated for this study are included in the article.

## Ethics Statement

The animal study was reviewed and approved by Animal Care and Use Committee of Hebei Medical University.

## Author Contributions

ZJ designed the study. SW, QG, LH, YM, YG, WZ, and PL performed the experiments. ZJ, SW, and HZ analyzed the data. ZJ, SW, PL, WZ, and HZ wrote the manuscript.

## Conflict of Interest

The authors declare that the research was conducted in the absence of any commercial or financial relationships that could be construed as a potential conflict of interest.
